# Image Quality and Stenosis Assessment of Non-Contrast-Enhanced 3-T Magnetic Resonance Angiography in Patients with Peripheral Artery Disease Compared with Contrast-Enhanced Magnetic Resonance Angiography and Digital Subtraction Angiography

**DOI:** 10.1371/journal.pone.0166467

**Published:** 2016-11-18

**Authors:** Jiayi Liu, Nan Zhang, Zhaoyang Fan, Nan Luo, Yike Zhao, Xiaoming Bi, Jing An, Zhong Chen, Dongting Liu, Zhaoying Wen, Zhanming Fan, Debiao Li

**Affiliations:** 1 Department of Radiology, Beijing Anzhen Hospital, Capital Medical University, Chaoyang District Anzhen Road 2nd, Beijing, 100029, China; 2 Biomedical Imaging Research Institute, Cedars-Sinai Medical Center, Los Angeles, California, United States of America; 3 Siemens Healthcare, China, MR Collaborations NE Asia, Beijing, China; 4 Department of Vascular Surgery, Beijing Anzhen Hospital, Capital Medical University, Chaoyang District Anzhen Road 2nd, Beijing, 100029, China; Bascom Palmer Eye Institute, UNITED STATES

## Abstract

**Purpose:**

To evaluate the diagnostic performance of flow-sensitive dephasing (FSD)-prepared steady-state free precession (SSFP) magnetic resonance angiography (MRA) at 3 T for imaging infragenual arteries relative to contrast-enhanced MRA (CE-MRA) and digital subtraction angiography (DSA).

**Materials and Methods:**

A series of 16 consecutive patients with peripheral arterial disease (PAD) underwent a combined peripheral MRA protocol consisting of FSD-MRA for the calves and large field-of-view CE-MRA. DSA was performed on all patients within 1 week of the MR angiographies. Image quality and degree of stenosis was assessed by two readers with rich experience. Inter-observer agreement was determined using kappa statistics. Receiver operating characteristic (ROC) curve analysis determined the diagnostic value of FSD-MRA, CE-MRA, and CE-MRA combined with FSD-MRA (CE+FSD MRA) in predicting vascular stenosis.

**Results:**

At the calf station, no significantly difference of subjective image quality scores was found between FSD-MRA and CE-MRA. Inter-reader agreement was excellent for both FSD-MRA and CE-MRA. Both of FSD-MRA and CE-MRA carry a stenosis overestimation risk relative to DSA standard. With DSA as the reference standard, ROC curve analysis showed that the area under the curve was largest for CE+FSD MRA. The greatest sensitivity and specificity were obtained when a cut-off stenosis score of 2 was used.

**Conclusion:**

In patients with severe PAD,3 T FSD-MRA provides good-quality diagnostic images without a contrast agent and is a good supplement for CE-MRA. CE+FSD MRA can improve the accuracy of vascular stenosis diagnosis.

## Introduction

Patients with peripheral artery disease (PAD) are prone to several quality-of-life impairing conditions, such as intermittent claudication, pain at rest, and even gangrene[1]. PAD symptoms can often be improved by intervention therapy or surgery. It is important that anatomic localization and stenosis degree assessmentbe performed before proceeding with a PAD management course[2]. Contrast-enhanced (CE)-magnetic resonance angiography (MRA) and computed tomography angiography (CTA) are well accepted as comprehensive assessment methodswith good accuracy for these pretreatment studies. However, these imaging methods are contraindicated in patients with renal insufficiency due to the risk of nephrogenic systemic fibrosis from exposure to gadolinium-based agents and contrast-induced acute kidney injury[3,4]. To avoid these risks, it is preferable that such patients instead be examined withnon-contrast-enhanced (NE)-MRA techniques.

Although several NE-MRA methods have been developed in recent years[5], their applications are limited bymotion artifactsproduced by relatively long acquisition timesas well as tendencies to overestimate the severity of low-grade to moderate stenosis[6,7]. The following NE-MRA alternatives to CE-MRA have been developed recently: Native SPACE (noncontrastangiography of the arteries andveins sampling perfection with application-optimized contrast by using different flip angle evolution)[3], quiescent interval single-shot (QISS)[6,8], and balanced steady-state free precession (SSFP) witha flow-sensitive dephasing (FSD) magnetization preparation [9]. Being a 3D fast spin-echo technique, Native SPACE enables images to be acquired at a high spatial resolution and is insensitive to static field inhomogeneity. But Native SPACE sequencesare highly susceptible to motion-related disruptions[10]. ECG-gated QISS MRA with SSFP performed at 3 T magnetismhas been shown to acquire credible angiographic images in the lower extremities[11–13]. QISS MRAhas the advantages of not requiringindividualized modification of imaging parameters andhaving a low sensitivity to motion. Conversely, 3 T QISS-MRAhas the disadvantage of segments sometimes yielding non-diagnostic image quality due to local field inhomogeneity [11] and parallel acquisition. Meanwhile, FSD-prepared SSFP MRA (FSD-MRA) is appreciated for enabling images to be acquired with isotropic submillimeter spatial resolution and its relatively high arterial blood signal-to-noise ratio and blood-tissue contrast-to-noise ratio.

With 1.5-T [9,14–16] and 3-T [17] MR systems, FSD-MRA has been shown to produce accurate results consistent with CE-MRA results. Notwithstanding, the performance of FSD-MRA relative to digital subtraction angiography (DSA) is unclear. Though FSD-MRA at 3T may increase the blood-tissue contrast-to-noise ratio (CNR), concerns remain regarding the propensity of balanced steady state free precession (bSSFP) sequences for off-resonance artifacts, which appear to worsen at 3 T [18].

Given the aforementioned considerations, the purpose of this study was to evaluate the diagnostic performance of FSD-MRA at 3T, relative to CE-MRA and DSA, in imaging infragenual arteries. We chose to focus on infragenual arteries in this study firstly because there is a need for non-contrast alternatives in the calf that will reduce venous contamination in conventional MRA, and secondly because it is particularly difficult to evaluate branches of calf arteries projecting in different directions in MRA.

## Materials and Methods

### Patient selection

This prospective study was approved by Anzhen Hospital Ethics committee. Sixteen consecutive patients (13 males, 3 females; mean age, 69.13 ± 12.73years; range 14–84 years) with symptoms of PAD who were referred to our department for peripheral CE-MRAs from July 2014 to July 2015 were included in this study (Table 1). All participants affirmed that they understood the nature of the study and signed informed consent forms before enrollment.

**Table 1:** Patient characteristics.

Characteristic	Value^a^
Mean age (age range), years	69.13 ± 12.73 (14–84)
Sex (males/females)	13/3
Mean body mass index, kg·m^-2^	25.75 ± 3.99
No. (%) smokers	12 (75.0%)
Mean no. cigarettes/day for smokers	15.83 ± 5.10
Duration of smoking for smokers, years	38.33 ± 14.03
Systolic blood pressure (mmHg)	127.00 ± 20.02
Diastolic blood pressure (mmHg)	84.00 ± 13.93
*Comorbidities and complications*, *no*. *(%) patients*	
Hyperlipidemia	8 (50.0%)
Hypertension	7 (43.6%)
Diabetes	7 (43.6%)
Coronary heart disease	15 (93.8%)
Cerebral infarction	10 (62.5%)
Intermittent claudication	15 (93.8%)
Rest pain	4 (25.0%)
Gangrene	3 (18.8%)
Mean (range) claudicatory distance, m	24 7.33 ± 215.89 (10–1000)

^a^Continuous data are presented as means ± standard deviations and categorical data as numbers and percentages.

### Magnetic resonance angiography

MRAs were performed on a 3-T MR system (Verio, Siemens Healthcare, Erlangen, Germany) with ECG-triggering. Each patient lay in a supine position (feet towards the imaging system), with a six-element body matrix coil and a 24-element peripheral angiography matrix coil combined with a spine coil for signal reception. FSD-MRA was performed first at the calf station, before administration of intravenous contrast material. Detailed technical parameters of FSD-MRA can be found elsewhere[9,16,17]. A CE-MRA protocol was employed with a 3D gradient-echo fast low-angle shot (FLASH) sequence; images were acquired at three stations in coronal orientation before and after contrast medium injection. The pulse sequence parameters used are summarized in Table 2.

**Table 2:** Pulse sequence parameters.

Parameter	CE-MRA	FSD-MRA
Parallel imaging factor	2	2
Acquisition time, s	63^a^	240–300^b^
Slice thickness, mm	0.9	1.0
Field of view, mm	350 × 400	400 × 320
Repetition time, ms	3.2	450
Echo time, ms	1.2	1.9
Flip angle, degrees	25	53
Matrix	369 × 448	288 × 294
Bandwidth, Hz per pixel	698	965

^a^19 s each sequence, plus 6 s for each table movement.

^b^Acquisition time dependent on heart rate; therefore, a range is provided.

For all examinations, contrast agent arrival to the abdominal aorta was detected with a 2D gradient-echo sequence. A bolus of Magnevist (0.1 mmol/kg bodyweight, up to maximum dose of 20 ml; Bayer Schering Pharma AG, Germany) was injected into the median cubital vein followed by a 20-ml saline flush (both injected at 2.0 ml/s).

### Conventional intra-arterial angiography

An experienced vascular surgeon performed all DSA examinations with a digital subtraction system (3100; GE Medical Systems, American). After insertion of a 5-Fr sheath (or 6 Fr-sheath if angioplasty was planned) through the common femoral artery contralateral to the lower limb with stenosis, a 4-Fr pigtail angiographic catheter was inserted into the lower abdominal aorta for aortoiliac DSA. Selective DSA was performed from the thigh to the foot. Appropriate injections were applied via a power injector at each station at a rate of 4–7-mL/s, depending on arterial flow. For the general procedure, a total of 40–60 mL of nonionic iodinated contrast agent (Iopamidol 370, Bracco, Shanghai, China) was used. Selective catheterization was performed whenever possible to improve vascular opacification.

### Image analysis

The quality of the FSD-MRA images was rated independently by two readers (J.Y.L. and N.Z., with 15 years and 5 years of experience in vascular imaging, respectively) who were blinded to the patients’ clinical histories and DSA results. Image data postprocessing and assessment were conducted at a Siemens workstation (SyngoMMWP VE40A, Siemens Med Service Software). Maximum intensity projection (MIP) images of the subtracted datasets were produced for assessment to enable comparisons with DSA results.

Five arterial segments were evaluated in each calf, including the popliteal artery, the tibiofibular trunk, the anterior tibial artery, the posterior tibial artery, and the fibular artery. Image quality and stenosis degree were assessed in all images. A 4-point scale was used to evaluate the image quality of each segment according to the following definitions: 1, poor (major arteries were unclear or had severe venous contamination); 2, fair (major arteries had some venous contamination); 3, good (major arteries had minor venous contamination); and 4, excellent (major arteries had no venous contamination)[9]. Segments that were given scores of 2, 3, or 4 were considered diagnostic. Image quality was not assessed for occluded segments because the lumen could not be seen[19].

The American College of Radiology grading system for peripheral MRA[20] was used for arterial stenosis degree assessment. Stenosis degree was assessed separately inFSD-MRA and CE-MRA images by the two aforementioned radiologists (J.Y.L. and N.Z.) after a 2-week hiatus. The accuracy of stenosis assessment based on FSD-MRA was evaluated relative to that based on CE-MRA and DSA, the reference standards in all 16 cases. Finally, the two aforementioned radiologists read FSD-MRA and CE-MRA images for each patient together, and made a final diagnosis for every arterial segment during MRA examination, and recorded as CE+FSD MRA.

The degree of stenosis revealed by DSA was determined by a vascular surgeon (Z.C.) with 30 years of experience in vascular surgery and DSA. Both FSD-MRA and CE-MRA were evaluated relative to DSA in all patients. If there were multiple narrow lesions present in thesame segment, the most severe lesion was used to describe the stenosis degree of the arterial segment [16].

### Statistical analysis

The quality of FSD-MRA versus CE-MRA images was compared with paired *t*-tests. Stenosis degree analysis results for FSD-MRA, CE-MRA, and DSA were also compared with paired *t*-tests. Inter-observer agreement was determined by kappa statistics. Sensitivity and specificity for detection of clinically significant (i.e. ≥50%) stenosis were calculated on a per segment basis and a per calf basis for FSD-MRA and compared to reference CE-MR values. Receiver operating characteristic (ROC) curve analysis was used to determine the cut-off values for FSD-MRA, CE-MRA, and FSD+CE-MRA that were most frequently associated with stenosis degree, as describedin the American College of Radiology grading system.The cut-off value was set to maximize the sum of sensitivity and specificity, which maximizes the difference between the sensitivity of the prognostic factor and the sensitivity that the prognostic factor would have if it did no better than random chance. Statistical significance was accepted at *p* ≤ 0.05. The statistical analysis was conducted on a segmental basis in SPSS 19.0 software (SPSS Inc., USA).

## Results

### Patient selection and image quality

The demographic characteristics of, cardiovascular risk factors of, and complicationsobserved in the patient cohortare reported in Table 1. None of the patients hada history of arrhythmia or renal failure (eGFR < 30 mL/min/1.73 m). All MRA and DSA examinations were completed successfully without any adverse events.

Images obtained from a total of 160 arterial segments (from 32 calves in 16 patients) were subjected to quality assessment. Vascular occlusion or motion artifacts prevented CE-MRA image quality assessment by Reader1 in30 of the 160 vessel segments (18.8%) and by Reader 2 in 31 of the 160 vessel segments (19.4%). Meanwhile, among theFSD-MRAsegment images,35/160(21.9%) and 37/160 (23.1%) existedocclusive lesions or non-diagnostic imagesby Reader 1 and Reader 2, respectively. All 16patients receivedDSAexaminations of 5 segments each, resulting in stenosis assessments of 80 segments. Overall, mean image quality did not differ between CE-MRA and FSD-MRA for either Reader 1 (FSD-MRA 3.63 ± 0.56 *vs*. CE-MRA 3.54 ± 0.78, *p* = 0.75) or Reader 2 (3.70 ± 0.55 *vs*. 3.60 ± 0.75, *p* = 0.82). Inter-reader agreement was excellent for both FSD-MRAand CE-MRA (results and statistical values are reported in Table 3).

**Table 3:** Comparison of image quality ratings and inter-observer agreement (Kappa).

Examination	Image quality ratings		Kappa
	Reader 1	Reader 2	
FSD-MRA	3.63 ± 0.56	3.70 ± 0.55	.898
CE-MRA	3.54 ± 0.78	3.60 ± 0.75	.877
*P* value	0.75	0.82	-

**Fig 1:**
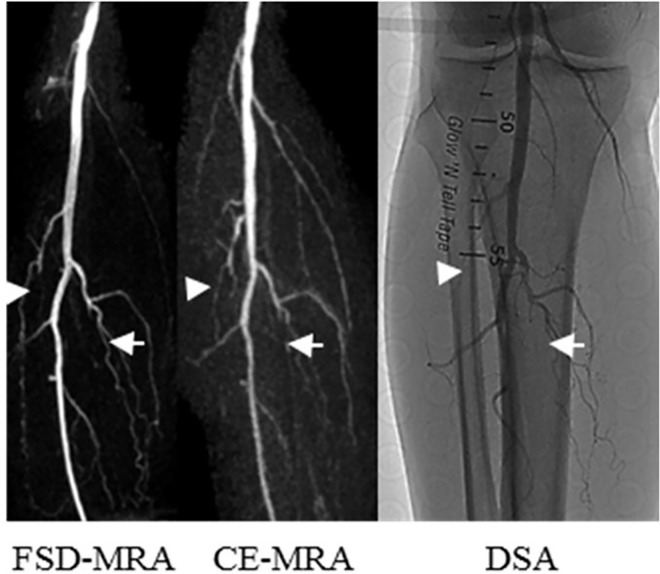
Images from a 64-year-old male patient who suffered from intermittent claudication for 8 years and rest pain for 1 month. Consistent with CE-MRA and DSA, FSD-MRA revealed an occlusive lesion on the right anterior tibial artery (white arrow) and right posterior tibial artery (white arrowheads) with collateral artery formation.

**Fig 2:**
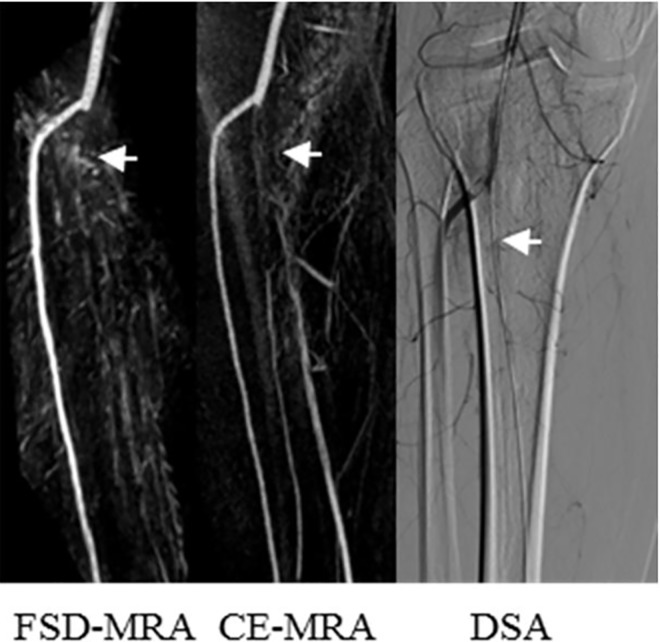
Images from a 37-year-old male patient who had been suffering from intermittent claudication for 2 weeks. Consistent with CE-MRA and DSA, FSD-MRA demonstrated an occlusive lesion on the right tibiofibular trunk (white arrow). Due to inhomogeneous background suppression, the distal right posterior tibial artery and right fibular artery are not shown clearly in the FSD-MRA image.

**Fig 3:**
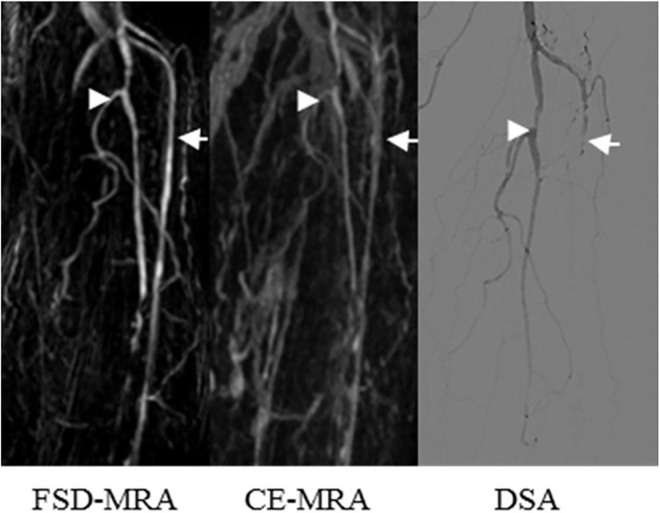
Images from an 81-year-old female patient with a 20-year history of diabetes suffered intermittent claudication for 1 year without rest pain. Consistent with CE-MRA and DSA, FSD-MRA revealed an occlusive lesion on the left posterior tibial artery (white arrowheads) and multiple significant stenosis lesions on the left tibiofibular trunk and left fibular artery. Venous contamination mimicked the left anterior tibial artery in FSD-MRA and CE-MRA, however an occlusion was apparent at that location in DSA (white arrow).

A representative example of superior venous suppression inFSD-MRA at the calf station is shown in Fig 1. Inhomogeneous background suppression in FSD-MRA (example in Fig 2) was encountered in 4/16 (25%) patients. Venous contamination in CE-MRA (example in Fig 3) was encountered in 5/16 (31%) patients. Off-resonance artifacts generated in the frequency-encode directionwere noted initially in the cranial and caudal portions of all 16 patients’ scans, but they could be removed from the region of interest by manual adjustment of the field of view (diameter, 275.9 ± 29.7 mm).

### Stenosis detection

Reader 1 excluded 4/160 segments (2.5%) from stenosis evaluation in CE-MRA due to inadequate image quality; no segments were similarly excluded by Reader 1 in FSD-MRA. Reader 2 excluded the same4/160 segments (2.5%) in CE-MRA that Reader 1 eliminated;1 segment (0.625%) was excluded by Reader 2 inFSD-MRA. Referencing the CE-MRA data as the standard, both readersdetermined that51 segments (Reader 1: 51/159, 32.1%; Reader 2: 51/156, 32.7%) had clinically significant (≥50%) stenosis. The disease burden was most severe in anterior tibial artery segments (both readers: 20/32, 62.5%) and posterior tibial artery segments (Reader 1: 14/32, 43.8%; Reader 2: 15/32, 46.9%).

The diagnostic accuracy data for FSD-MRA relative to CE-MRA for each reader are reported in Table 4. A consensus between FSD-MRA, CE-MRA, and CE+FSD MRA datasets was achieved for the two readers in all cases. Stenosis degree findings were similar for the three compared imaging methods (FSD-MRA, 1.68 ± 1.65; CE-MRA, 1.68 ± 1.57; DSA, 1.48 ± 1.63; F_2,237_ = 0.407, *p* = 0.67). With respect to the diagnosis of individual segments, FSD-MRA had greater sensitivity, but less specificity, than CE-MRA (Table 5). At the level of whole calves, FSD-MRA had less sensitivity and less specificity than CE-MRA (Table 5).

**Table 4:** Diagnostic accuracy (95%CI) of FSD-MRA, relative to CE-MRA, for detection of clinically significant (≥50%) stenosis.

*Level*, reader	Sensitivity	Specificity	PPV	NPV
*Segment*				
Reader 1	0.840(0.703–0.923)	0.950(0.880–0.978)	0.875(0.741–0.948)	0.929(0.860–0.966)
Reader 2	0.854(0.716–0.935)	0.944(0.878–0.977)	0.872(0.736–0.947)	0.936(0.868–0.972)
*Calf*				
Reader 1	0.917(0.715–0.985)	0.875(0.467–0.993)	0.957(0.760–0.998)	0.778(0.402–0.961)
Reader 2	0.917(0.715–0.985)	0.750(0.356–0.955)	0.917(0.715–0.985)	0.750(0.356–0.955)

**Table 5:** Diagnostic accuracy (95%CI) of FSD-MRA and CE-MRA, relative to DSA as a reference standard.

*Level*, method	Sensitivity	Specificity	PPV	NPV
*Segment*				
FSD-MRA	0.862(0.674–0.955)	0.863(0.731–0.938)	0.781(0.596–0.901)	0.917(0.791–0.973)
CE-MRA	0.828(0.635–0.935)	0.902(0.778–0.963)	0.828(0.635–0.935)	0.902(0.778–0.963)
*Calf*				
FSD-MRA	0.923(0.621–0.996)	0.667(0.125–0.982)	0.923(0.621–0.996)	0.667(0.125–0.982)
CE-MRA	1.000(0.717–1.000)	1.000(0.310–1.000)	1.000(0.717–1.000)	1.000(0.310–1.000)

**Fig 4:**
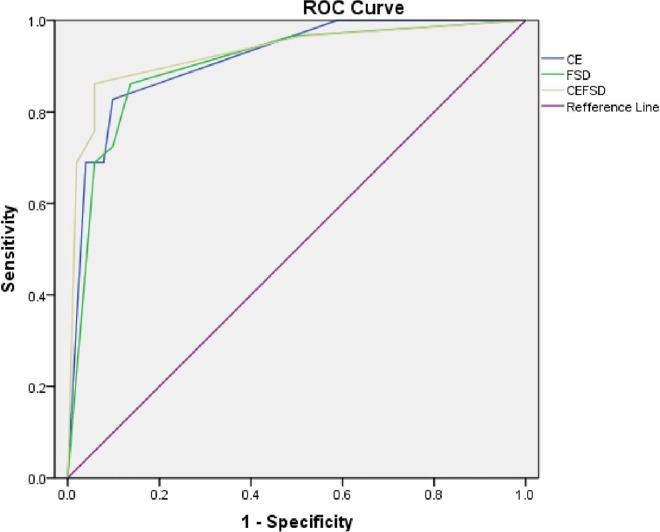
ROC curves of stenosis degree detection in FSD-MRA, CE-MRA, and CE+FSD-MRA with DSA as the standard reference. The area under the curve was largest for CE+FSD MRA (yellow, 0.929, *p* < 0.01), and was slightly larger for CE-MRA (blue, 0.915, *p* < 0.01) than for FSD-MRA (green, 0.903, *p* < 0.01).

ROC curve analysis, with DSA as the standard reference, yielded asensitivity/specificity maximizing cut-off stenosis degree value of 2 for FSD-MRA, CE-MRA, and CE+FSD-MRA.The area under the ROC curve was largest for CE+FSD MRA (0.929, *p* < 0.01). The area under the ROC curve was larger for CE-MRA (0.915, *p* < 0.01) than FSD-MRA (0.903, *p* < 0.01) (Fig 4, Table 6). With stenosis degree cut-off value of 2, CE+FSD MRA had a sensitivity value similar to that of FSD-MRA (86.2%), but highest in specificity value (94.1%).

**Table 6:** Diagnostic accuracy of FSD-MRA, CE-MRA, and CE+FSD-MRA for detection of clinically significant stenosis, relative to DSA, with a stenosis degree cut-off value of 2.

Modality	Sensitivity	1-Specificity	Specificity	Sensitivity+Specificity
CE-MRA	0.828	0.098	0.902	1.730
FSD-MRA	0.862	0.137	0.863	1.725
CE+FSD MRA	0.862	0.059	0.941	1.803

A total of 69 segments were scored 3 and 4. Stenosis degree of CE MRA was 1.58 ± 1.59, FSD MRA 1.52 ± 1.67, CE+FSD MRA 1.54 ± 1.63, and DSA 1.42 ± 1.62. The area under the ROC curve for CE+FSD MRA, CE MRA, and FSD MRA were 0.929 ± 0.038, 0.913 ± 0.036, and 0.911 ± 0.040. Sensitivity (87.0%) and specificity (93.5%) of FSD+CE MRA in these 69 segments scored 3 and 4 were highest. CE MRA had a specificity value similar to that of CE+FSD MRA (93.5%). CE MRA had lowest sensitivity (78.3%), meanwhile FSD MRA had lowest specificity (91.3%) using segments with scores 3 and 4. The constant results were combined MRA protocol can increase diagnostic accuracy.

## Discussion

Nonenhanced MRA is a promising alternative to enhanced MRA for evaluation of calf arteries. Previously, Knobloch et al.[21] examined the image quality and diagnostic performance of QISS and Native Space sequences using CE MRA as a standard. The results indicated that the QISS sequence at 3 T was a robust nonenhanced MRA technique. However, 3-T QISS-MRA has the disadvantage of segments sometimes yielding a non-diagnostic image quality due to local field inhomogeneity[11] and parallel acquisition. Meanwhile, FSD-prepared SSFP MRA (FSD-MRA) has the advantage of enabling images to be acquired with isotropic submillimeter spatial resolution together with a relatively high arterial blood signal-to-noise ratio and blood-tissue contrast-to-noise ratio. In the present study, we compared the performance of FSD MRA and CE MRA of calf arteries for a definitive diagnosis of luminal stenosis and found that, relative to prior work in which CE MRA was used as the standard[17], we obtained more accurate assessments employing DSA as the standard. To offset the disadvantages of FSD MRA and CE MRA, we adopted a combined MRA protocol, which when applied with ROC curve analysis, can increase diagnostic accuracy. The present study demonstrated that 3-T FSD-MRA produces credible diagnostic images of infragenual arteries, yielding results that are consistent with DSA results. Overall mean image quality, as determined by two expert readers, was similar between CE-MRA and FSD-MRA. Inter-reader agreement was excellent for both FSD-MRA and CE-MRA scans. Our analysis of the diagnostic accuracy of FSD-MRA, CE-MRA, and CE+FSD MRA, with DSA as the standard reference, indicated that the sensitivity of 3-T FSD-MRA for detecting significant stenosis within a vessel segment was acceptable, and marginally higher than that of CE-MRA. However, specificity was lower for FSD-MRA than for CE-MRA. Stenosis sensitivity and specificity for FSD-MRA at 3 T were slightly better than values obtained previously with FSD-MRA at 1.5 T[14].

The main factors influencing the diagnostic accuracy of FSD-MRA include changes in arterial blood flow direction, inhomogeneous background suppression caused by motion artifacts, and off-resonance artifacts[16,17]. These issues can lead to overestimation of stenosis degree and reduced specificity of FSD-MRA. There is a risk of overestimating the degree of stenosis in the anterior tibial arteries, especially near the vessel curvature, because of arterial flow changes.

Although CE-MRA has good sensitivity and specificity, venous contamination can lead to false positives in which radiologists mistake normal arteries for occluded or severely stenosed arteries[22]. Because venous contamination in CE-MRA can result in some segments being non-diagnostic, we use FSD MRA in this study to improve the diagnostic accuracy in segments with venous contamination. Use of FSD-MRA can eliminate venous contamination in calf arteries, producing excellent artery delineation. Importantly, FSD-MRA and CE-MRA findings were found to be highly consistent in our study. The shortcomings of CE-MRA and FSD-MRA could be overcome with CE+FSD MRA, which yielded superior specificity, a critical parameter of accuracy. Our results indicate that FSD-MRA can be used in place of CE-MRA in patients with contraindications to gadolinium. Furthermore, our results indicate that FSD-MRAis also a good supplementary means of improving the accuracy of CE-MRA.

Although the FSD-MRA sequence was performed successfully, we observed several limitations. Firstly, inhomogeneous background suppression and off-resonance artifacts reduced FSD-MRA image quality. The inhomogeneous background suppression can be attributed to motion-related artifacts due to the relatively long acquisition time of the method. The duration of the procedure was further prolonged by the need for shimming and blood-flow parameter measurement taking. Meanwhile, off-resonance artifacts arise from increased susceptibility effects that cannot be avoided in bSSFP images at 3 T. Susceptibility to magnetic field inhomogeneity has limited the wide application of SSFP sequence at 3 T. This issue may be exacerbated in peripheral scans with limited fields of view. In our study, shimming of the main magnetic field was employed before each vascular acquisition scan to reduce these effects. Secondly, this study was performed in a small sample and included several vascular segments that were not well assessed by FSD-MRA or CE-MRA. Thirdly, the possibility that the two blinded observers might have been able to identify sequence characteristics from the images represents a potential source of bias. Despite these limitations, our findings support the notion that FSD-MRA is a promising technique with notably high sensitivity for stenosis detection. FSD-MRA represents a potential alternative tool for patients with contraindications to gadolinium contrast media. Future studies should have: (1) a large number of patients with DSA as a reference; (2) whole lower extremity FSD MRA imaging; and (3) reduced FSD MRA scanning time and motion artifacts.

In conclusion, 3-T FSD-MRA can acquire good-quality diagnostic images without a contrast agent in patients with severe PAD. Therefore, it has the potential for application in patients with contraindications to gadolinium. Additionally, FSD-MRA represents a good supplement to CE-MRA, and CE+FSD MRA can improve the accuracy of vascular stenosis diagnosis.
